# Nuclear Localization of G3BP6 Is Essential for the Flowering Transition in *Arabidopsis*

**DOI:** 10.3390/biom13121697

**Published:** 2023-11-24

**Authors:** Yuzhu Wang, Zhiyong Li, Xiaoju Liang, Yeling Zhou, Jiansheng Liang

**Affiliations:** 1 Jiangsu Key Laboratory of Crop Genetics and Physiology/Co-Innovation Center for Modern Production Technology of Grain Crop, Yangzhou University, Yangzhou 225009, China; 2 Department of Biology, Southern University of Science and Technology, Shenzhen 518055, China; 3 Key Laboratory of Molecular Design for Plant Cell Factory of Guangdong Higher Education Institutes, Department of Biology, Southern University of Science and Technology, Shenzhen 518055, China; 4 Academy for Advanced Interdisciplinary Studies, Southern University of Science and Technology, Shenzhen 518055, China; 5 College of Life Sciences, Fujian Agriculture and Forest University, Fuzhou 350002, China

**Keywords:** *Arabidospis*, G3BP, subcellular localization, flowering transition, miRNA156-SPL

## Abstract

The Ras GTPase-activating protein SH3 domain-binding protein (G3BP) belongs to the highly conserved family of RNA-binding proteins, which has been well-investigated in humans and animals. However, limited study of plant G3BP has been reported, and the precise biological function of the G3BP family has not been elucidated yet. In this study, the *Arabidopsis* G3BP family, comprising seven members, was comparatively analyzed. Transcriptome analysis showed that most *G3BP* genes are ubiquitously expressed in various tissues/organs. Transient expression analysis revealed that all G3BPs were presented in the cytoplasm, among which G3BP6 was additionally found in the nucleus. Further study revealed a conserved NLS motif required for the nuclear localization of G3BP6. Additionally, phenotypic analysis revealed that loss-of-function *g3bp6* presented late-flowering phenotypes. RNA-sequencing analysis and qRT-PCR assays demonstrated that the expressions of abundant floral genes were significantly altered in *g3bp6* plants. We also discovered that overexpression of G3BP6 in the nucleus, rather than in the cytoplasm, propelled bolting. Furthermore, we revealed that the scaffold protein Receptor for Activated C Kinase 1 (RACK1) interacted with and modulated the nuclear localization of G3BP6. Altogether, this study sheds new light on G3BP6 and its specific role in regulating the flowering transition in *Arabidopsis*.

## 1. Introduction

The Ras GTPase-activating protein SH3 domain-binding protein (G3BP) was firstly identified as a Ras-GAP binding protein and later shown to function as an endoribonuclease that selectively targets genes by binding to their consensus sequences [1,2]. G3BPs consist of several conserved domains. The N-terminus is characterized by a nuclear transport factor 2 (NTF2)-like domain, which is suggested to participate in the nuclear transport of proteins driven by nuclear location signals (NLSs) and facilitates protein–protein interactions. At the C-terminus, there is an RNA recognition motif (RRM) followed by an arginine- and glycine-rich region (RGG domains) [3,4,5]. The presence of the RGG motif suggests that G3BP is primarily an RNA-binding protein (RBP), which enhances its binding affinity to and plays a role in the nucleocytoplasmic shuttling of RNA [6,7,8].

G3BPs belong to the highly conserved protein family, which has been well-investigated in human and animals. G3BPs have been shown to participate in the regulation of multiple cellular functions, including mRNA stability, stress granule (SG) formation, virus replication, DNA pattern recognition and tumor signaling transduction [9,10,11]. G3BPs are also well-known to play an important role in viral immunity, since SGs are the primary target of virus infection [12]. In the model plant *Arabidopsis*, seven G3BP-like proteins have been identified with both the NTF2 and RRM domains [13,14]. This also shows that all plant G3BPs, like human and animal G3BPs, promote SG formation. A G3BP-like protein in *Arabidopsis* was identified as related to SG formation and played a role in virus resistance [14]. *Arabidopsis* G3BP orthologues can rescue mammalian SG formation, suggesting a general pathway of SG formation over living organisms [15]. However, at present, little is known about the precise biological functions of G3BP-family proteins in regulating plant growth and development as well as in plant responses to biotic/abiotic stresses.

In this study, we found that G3BP6, one of the G3BP family from *Arabidopsis*, plays a key role in regulating the flowering transition. The *g3bp6* mutant showed a significantly delayed flowering phenotype, indicating that G3BP6 positively controls *Arabidopsis* flowering. RNA-sequencing analysis and a qRT-PCR assay demonstrated that G3BP6 affected the abundance of floral genes, partially through the regulatory network of the miR156-SPLs module. Interestingly, we found that overexpression of G3BP6 in the nucleus but not in the cytosol effectively regulates the expression of flowering-associated genes and promotes bolting. Additionally, our results show that the scaffold protein RACK1 (Receptor for Activated C Kinase 1) interacted with G3BP6 and modulated the nuclear localization of G3BP6. Collectively, this study reveals a specific role of G3BP6 in the regulation of the *Arabidopsis* flowering transition, providing new insights into the function of G3BPs in plants.

## 2. Materials and Methods

### 2.1. Plant Materials and Growth Condition

A wild-type *Arabidopsis thaliana* Col-0 accession was used in this study. The mutants used in this study, including *g3bp1* (SALK_027468C), *g3bp2* (SALK_204332C), *g3bp3* (SALK_038222C), *g3bp4* (SALK_023921C), *g3bp5* (SALK_206730C), *g3bp6* (SALK_042146C) and *g3bp7* (SALK_019963C), were obtained from the Arabidopsis Community Arashare (www.arashare.cn, accessed on 15 June 2021). The mutant *rack1a-2* [16] was crossed with *gb3bp6* to generate double-mutant *rack1a gb3bp6*. The plant growth was carried out in a culture room at 22 °C over a long-day photoperiod (16 h:8 h; light:dark, respectively). The individual T-DNA insertion line was confirmed by genotyping and sequencing using the gene-specific primers shown in Table S1.

### 2.2. Subcellular Localization of G3BPs and Plant Transformation

For subcellular localization, the coding region of the individual *G3BP*, lacking the stop codon, was cloned into pUC35S-mCherry to generate *35S:G3BP-mCherry*, which was transformed into *Arabidopsis* protoplasts, as has been described previously [17]. The transient expression of the fusion protein was examined using a confocal laser-scanning microscope (Leica SP8; Leica Microsystems GmbH, Solms, Germany) at 24 h after transformation. The transformed protoplasts were excited with 552 nm, and their emission was detected in the range of 570–620 nm. The pinholes were adjusted to 1 Airy unit for each wavelength. Post-acquisition image processing, such as channel merging and the addition of scale bars, was performed with ImageJ (Version ij153-win-java8).

To generate the plasmids for the overexpression of G3BP6 and two other forms with either the nuclear localization signal (NLS) or nuclear export signal (NES) peptide in plants, the corresponding code region of G3BP was cloned into pDONOR221 and then recombined with the Gateway-compatible binary vector pGWB405. All binary vectors were sequenced and introduced into *Agrobacterium tumefaciens* strain GV3101 cells. Transgenic lines were generated with the Agrobacterium tumefaciens-mediated floral dip method [18]. All transgenic lines used in this study were homozygous T3 lines. The primers used for plasmid construction are listed in Table S1.

### 2.3. Analysis of Gene Expression with Quantitative Real-Time PCR (qRT-PCR)

The total RNA was isolated from the indicated tissues and organs in Col-0, *g3bp6* mutant and G3BP6 overexpression plants. The first-strand cDNA was synthesized using NovoScript^®^Plus All-in-one 1st Strand cDNA Synthesis SuperMix (Novoprotein Scientific Inc., Shanghai, China). qRT-PCR was performed on Step-one PlusTm from Applied Biosystems (Thermo Fisher Scientific Inc., Waltham, MA, USA). The transcript data were calculated with 2^−ΔΔCt^ to quantify the gene expression levels [19]. The *Actin2* gene was used as the internal control. Each experiment was performed with three replicates. The primers for the qRT-PCR are listed in Table S1.

### 2.4. Yeast Two-Hybrid and Bimolecular Fluorescence Complementation Assays

For the yeast two-hybrid (Y2H) assays, the coding region of RACK1 was cloned into the pGBKT7 vector to generate BD-RACK1, and the indicated regions of G3BP6 were cloned into the pGADT7 vector. The Y2H assays were performed following the manufacturer’s instructions from Clontech (Takara Bio Inc., Shiga, Japan). The transformed yeast cells were cultured on SD/-LT and SD/-LTHA media at 30 °C for 3 days. SD/-LTHA was the yeast culture medium without leucine, tryptophan, histidine or adenine.

For the bimolecular fluorescence complementation (BiFC) analysis, the coding region of RACK1 lacking the stop codon was cloned into pZY101-nYFP [20] to generate RACK1-nYFP. The coding regions of G3BP6 and G3BP7 were cloned into pZY101-cYFP to generate G3BP6-cYFP and G3BP7-cYFP, respectively. The resulting constructs were transformed into *A. strain* GV3101 cells and co-infiltrated into *N. benthamiana* leaves. YFP fluorescence was captured using a confocal laser scanning microscope (Leica SP8; Leica Microsystems GmbH, Solms, Germany) at 48 h after infiltration. The PCR primers used for these assays are listed in Table S1.

### 2.5. Transcriptome Analysis

For transcriptome (RNA-sequencing) analysis, 4-week-old Col-0, *g3bp6* and *g3bp7* plants were collected. The RNA sequencing was conducted by Genedenovo Biotechnology Co., Ltd. (Guangzhou, China). The cDNA libraries were sequenced on the Illumina sequencing platform. The raw sequence reads were uploaded to the Sequence Read Archive, and the code number was PRJNA975320. Significantly differentially expressed genes (DEGs) were defined using DESeq2 [21] with a twofold expression difference with a *p* value of <0.05.

## 3. Results

### 3.1. Expressional Patterns and Subcellular Localization of G3BPs in Arabidopsis

**Figure 1:**
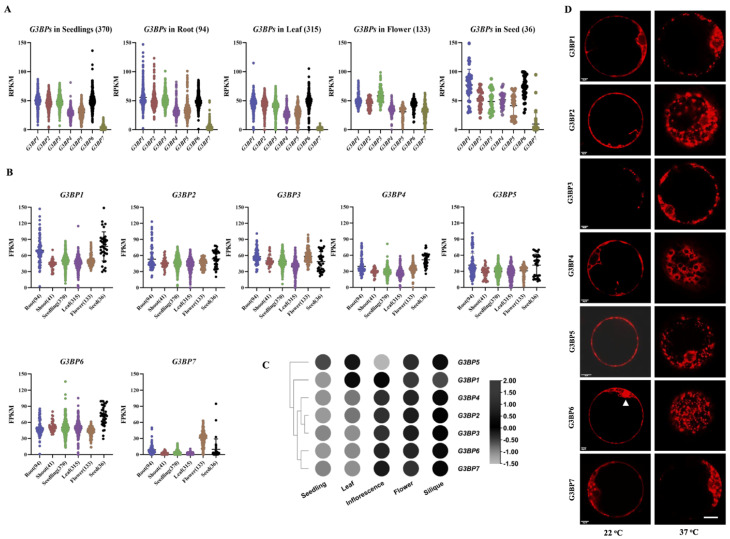
Expression pattern analysis of G3BP family in *Arabidopsis*. (**A**,**B**) Gene expression profiles for *G3BP* genes from selected tissues/organs based on analysis with transcriptome data retrieved from the public *Arabidopsis* RNA-seq Database. The number of sequencing libraries for different samples is indicated. RPKM: Reads Per Kilobase Million; FPKM: Fragments Per Kilobase Million. (**C**) Gene expression patterns of *G3BP* genes estimated with the qRT-PCR assay. The values were normalized to *AtACTIN2* and are expressed as means ± SD; *n*  =  3. (**D**) Transient expression analysis of G3BPs in *Arabidopsis* protoplasts. The growth chamber was subjected to heat stress treatment at 37 °C for 30 min before the confocal image analysis. The representative confocal images for the fluorescence of mCherry (in red color) are shown. The white triangle indicates the nucleus. Scale bar: 20 μm.

Previous studies have suggested functional similarities between human and *Arabidopsis* G3BPs [14,15]. To gain insights into the expression patterns of *G3BPs* in Arabidopsis, we analyzed the RNA-seq data from the intergraded *Arabidopsis* RNA-seq Database (http://ipf.sustech.edu.cn/pub/athrna/, accessed on 2 July 2022). The results showed that most *G3BP* genes manifested ubiquitous expression in all selected tissues or organs, except for *G3BP7*, which was exclusively expressed in flowers (Figure 1A,B). Higher expressions of most *G3BPs* in flowers were further verified with the qRT-PCR assay (Figure 1C and Figure S1). Although it has been reported that most plant G3BPs show similar subcellular localization in cells [12], the resolution of the cellular localization of G3BPs is still low in plant cells. In the present study, we expressed individual *G3BP* genes in *Arabidopsis* protoplasts and analyzed the details of the subcellular localization of the G3BPs in a single cell. Transient expression analysis revealed that most G3BPs presented in the cytoplasm, except that G3BP6 was additionally found in the nucleus (Figure 1D). It has been reported that all plant G3BPs promote SG formation [12]. We investigated if the transient expressions of different G3BPs in tobacco mesophyll cells would induce SG formation under heat shock stress. Indeed, the expressions of all the tested G3BPs in *Arabidopsis* protoplasts resulted in notable SG-like structures (Figure 1D). Additionally, it is interesting to note that both G3BP3 and G3BP7 had already formed SG-like structures under ambient conditions (Figure 1D).

### 3.2. The Nuclear Localization Signal (NLS) Mainly Determines the Nuclear Localization of G3BP6

**Figure 2:**
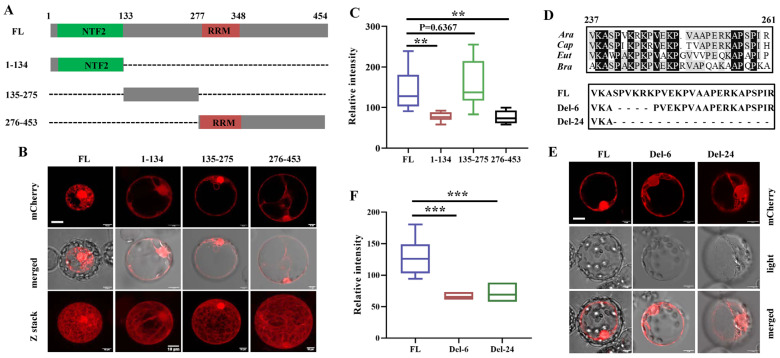
The subcellular localization of G3BP6 in *Arabidopsis* protoplasts. (**A**) Schematics of various truncated forms of G3BP6. The numbers indicate positions of amino acids. NTF2: nuclear transport factor 2-like domain; RRM: RNA recognition motif. (**B**) Representative images for the subcellular localizations of various G3BP6 variants from (**A**). Z-stack images were taken from the corresponding single cells. (**C**) Quantification of the signal intensity of mCherry fused with various G3BP6 truncated proteins from the nucleus in (**B**). (**D**) Schematic representation of the putative NLS (amino acids 241–256) and its mutants. Del-6: amino acids (241–246) deleted; Del-24: amino acids (241–261) deleted. (**E**) Subcellular localizations of the truncated G3BP6 variants within the NLS. (**F**) Quantification of the signal intensity of mCherry fused with these G3BP6 truncated proteins from the nucleus shown in (**E**). Error bars are indicated as means ± SE (*n* = 15) (** *p* < 0.01, *** *p* < 0.001, Student’s *t* test). Scale bar: 10 μm.

The NTF2-like domain has been suggested to play a role in the nuclear shuttling of G3BP [22,23,24]. Although the NTF2-like domain is present in all *Arabidopsis* G3BP homologues, previous studies and our own have indicated that only G3BP6 displays both cytoplasm and nuclear localization (Figure 1D). Here, we have further explored the roles of the NTF2-like domain in determining the nuclear localization of *Arabidopsis* G3BP6 by analyzing the subcellular localizations of various truncated G3BP6 fragments (Figure 2A). The results showed that all the truncated G3BP6 fragments, either with or without the NTF2-like domain, were still detected in the nucleus (Figure 2B). Moreover, the nuclear signal intensities of the two truncated G3BP6 fragments, one with the NTF2-like domain (1–134 aa) and one without the NTF2-like domain (276–453aa), transiently expressed in the *Arabidopsis* protoplasts were much lower than that of the full length (FL) or the truncated G3BP6 fragment (135–275 aa) (Figure 2B,C). This indicates that the expression of G3BP6 in the nucleus was not related to the NTF2-like domain. We then investigated whether the potential nuclear localization signal (NLS) was presented in the 135–275 aa sequence of G3BP6 using online NLS prediction software NLStradamus (Revision r.9 - Last Updated on: March 25 2020) [25]. The bioinformatic analysis showed the potential NLS in G3BP6 from numbers 241 to 256 of the amino acid sequence (KRKPVEKPVAAPERKA), which is relatively well-conserved in plant species such as Capsella, Eutrema and Brassica (Figure 2D). To evaluate the potential contribution of the NLS to the nuclear localization of G3BP6, two truncated versions of G3BP6 with the integrity of the NLS disrupted were designed for further analysis. It was revealed that the nuclear accumulation of G3BP6 was significantly decreased with the NLS sequence disrupted (Figure 2D–F). However, the nuclear localization of the G3BP6 was not completely abolished even when the assigned NLS was deleted, implying the involvement of other relevant factors in the nuclear shuttling of G3BP6.

### 3.3. g3bp6 Mutation Significantly Delays the Flowering Time

**Figure 3:**
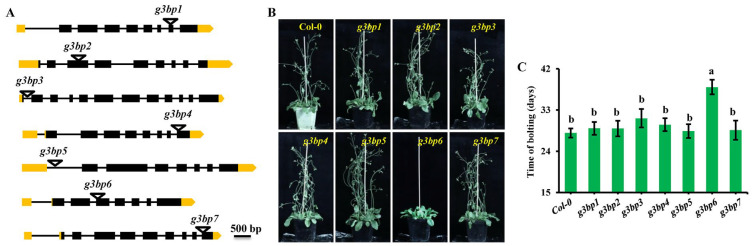
Characterizations of the *g3bp* mutants. (**A**) Schematic representation of the locations of the T-DNA insertions within the *G3BP* genes. Exons and introns are indicated with black boxes and lines, respectively, and the UTR region is indicated in yellow. White triangles denote the sites of T-DNA insertion. (**B**) Phenotypic analysis of wild-type and *g3bp* mutant plants grown under LD conditions for eight weeks. (**C**) Quantifications of the blotting time for wild-type Col-0 and *g3bp* mutants from (**B**). Three biological replications were performed and similar results were observed. The lowercase letters denote statistically significant differences between the indicated samples, as determined with one-way ANOVA (*p* < 0.05). Error bars are indicated as means ± SE (*n* = 12).

At the present, the roles of G3BPs in plant growth and development as well as in responses to environmental stresses are still largely unknown. To explore the roles of G3BPs in plant growth and stress responses, we first obtained the mutant lines for all seven G3BPs. Individual T-DNA insertion lines were verified, and the site of insertion was determined with sequencing. It was revealed that T-DNA is mostly inserted in either the exon- or intron-spanning code region (Figure 3A). Phenotypic analysis showed that most *g3bp* mutants had no visible differences in either growth or developmental events compared to the wild-type Col-0, except that the *g3bp6* mutant with the T-DNA inserted in the third exon exhibited a significantly delayed flowering phenotype (Figure 3B,C).

### 3.4. G3BP6 Controls Global Gene Expression with Specific Roles in the Flowering Transition

**Figure 4:**
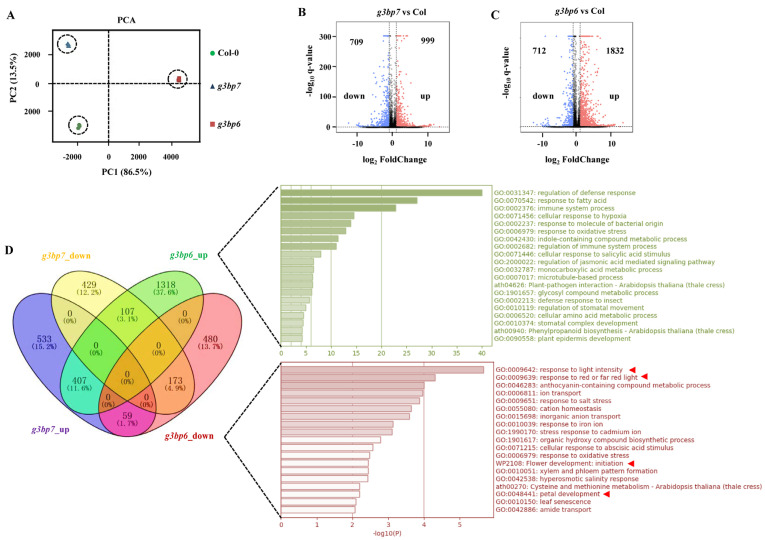
RNA-sequencing analysis of differential gene expression among Col-0, *g3bp6* and g3bp7. (**A**) PCA plot of RNA-seq data from Col-0 (circle), *g3bp6* (square) and *g3bp7* (triangle) samples. Each dot represents one sample; % variability encompassed within each principal component is shown. (**B**,**C**) Volcano plots of all genes showing differential expression. Pink and blue dots denote significant upregulation and downregulation, respectively. Blue dots indicate a decrease in value, pink indicates an increase, black indicates no change. (**D**) Gene ontology (GO) analysis of the most significantly enriched GO terms in the biological process from DEGs uniquely found in *g3bp6* based on comparison to the Venn diagram.

To explore the role of G3BP6 in controlling the flowering transition, we performed an RNA-sequencing experiment in the *g3bp6* mutant. As *G3BP7* is exclusively highly expressed in *Arabidopsis* flowers (Figure 1A–C) and no visible change in *G3BP6* transcription was detected in the *g3bp7* mutant (Figure 3C), we included the *g3bp7* mutant in the sequencing experiment as a control. Four-week-old plants with Col-0, *g3bp6* and *g3bp7* mutants were sampled and used for RNA-sequencing analysis. The principal component analysis (PCA) of these transcripts showed high correlations between the three biological replicates of the Col-0, *g3bp6* and *g3bp7* samples (Figure 4A). Totals of 2544 and 1708 differentially expressed genes (DEGs) (fold change ≥ 2, false discovery rate (FDR) < 0.01) were identified in the *g3bp6* and *g3bp7* mutants, respectively, compared with those in the Col-0 (Figure 4B,C). Among those DEGs, 1832 and 712 were upregulated and downregulated, respectively, in the *g3bp6* mutant, whereas 999 and 709 DEGs were upregulated and downregulated, respectively, in the *g3bp7* mutant. To better visualize the correlations of the DEGs in the *g3bp6* and *g3bp7* mutants, we plotted the common and unique genes using Venn diagrams. In total, 564 overlapping genes (representing 29.33% of the DEGs in *g3bp6* and 43.68% in *g3bp7*) were identified (Figure 4D), suggesting the diverse roles of G3BP6 and G3BP7.

The above data analysis revealed that 1318 and 480 DEGs were found to be specifically upregulated and downregulated, respectively, in *g3bp6* compared with in Col-0. These DEGs were further analyzed based on their gene ontology (GO). The enrichment analysis of this gene ontology revealed that the DEGs were mostly associated with plant responses to stress stimuli and plant development (Figure 4D). Notably, the strong enrichment observed in the downregulated DEGs (480 out of 712) in the *g3bp6* mutant was found to be related to the light response and flower organ development (Figure 4D).

**Figure 5:**
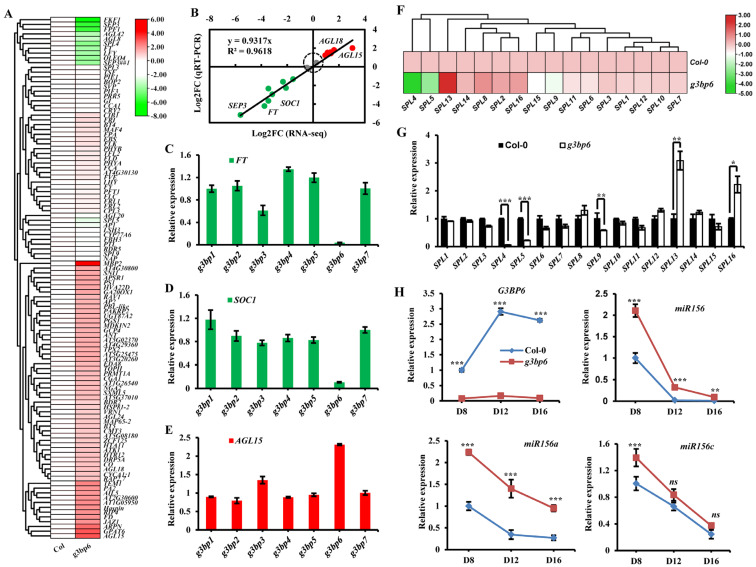
G3BP6 controls global gene expression in response to the flowering transition. (**A**) Heatmap showing differential expression genes related to flowering from Col-0 and *g3bp6* plants. (**B**) Verification of RNA-sequencing results with qRT-PCR analyses of 20 representative genes. The correlation coefficient (R^2^) is indicated in the figure. Red dots indicate a increase in value, green indicates an decrease, grey indicates no change. (**C**–**E**) Results of qRT-PCR analysis of related genes, including *FT* (**C**), *SOC1* (**D**) and *AGL15* (**E**), from Col-0 and *g3bp* mutants. Error bars indicate SEs (*n* = 4, four biological replicates). (**F**) Heatmap showing differential expression for all *SPL* genes from Col-0 and *g3bp6* plants. (**G**) Results of qRT-PCR analysis of *SPL* genes from wild-type and *g3bp6* mutants. Error bars indicate as means ± SE (*n* = 3) (* *p* < 0.05, ** *p* < 0.01, *** *p* < 0.001, Student’s *t*-test). (**H**) Results of qRT-PCR analysis of temporal variations of *G3BP6*, *miR156*, *pri-miR156a* and *pri-miR156c* levels in the shoot apices of Col and *g3bp6* mutant plants. Values are relative to Col-0 at day 8 and each represent the mean SE from three biological replicates. *** *p* < 0.001; one-way analysis of variance (ANOVA). ns: no significant difference.

To further evaluate the role of G3BP6 in the regulation of the *Arabidopsis* flowering transition, we first analyzed the expression patterns of flowering-related genes from Col-0 and the g3bp6 mutant. The results showed that, in a total of 109 selected floral genes, 18 of them were upregulated and 54 were downregulated in the *g3bp6* mutant (Figure 5A). We further randomly selected 20 DEGs and quantified their expression with qRT-PCR. The results were consistent with the RNA-sequencing analysis, with all the tested DEGs exhibiting similar trends (Figure 5B), implying the reliability of the RNA-sequencing data. Among these genes, the floral integrator FLOWERING LOCUS T (FT) and SUPPRESSOR OF CONSTANS 1 (SOC1) are the major targets of multiple flowering pathways [26,27,28]. Consistently, *FT* and *SOC1* were significantly downregulated in the *g3bp6* mutant as compared to either Col-0 or to any other *g3bp* mutant (Figure 5C,D). Furthermore, we found that two MADS-domain proteins, AGAMOUS-LIKE15 (*AGL15*) and *AGL18*, which act as floral repressors [29,30], were remarkably upregulated, accompanied by G3BP6 mutation (Figure 5A,E). Notably, the expression of several SQUAMOSA PROMOTER BINDING-LIKE (SPL) genes, which are involved in the control of flowering [31,32], were significantly altered in the *g3bp6* mutant as compared with those of Col-0 (Figure 5F). Specifically, the transcript levels of *SPL4*, *SPL5* and *SPL9* were substantially decreased in the *g3bp6* mutant in comparison with those in Col-0 (Figure 5G). Additionally, as *SPL4* and *SPL5* are also post-transcriptionally regulated by microRNA156 (miR156) during shoot development [33], we tested whether G3BP6 regulates *SPL* expression by modulating amounts of *miR156*. We used qRT-PCR to examine the levels of *miR156* in the shoot apices of the Col-0 and *g3bp6* plants at 8, 12 and 16 d after planting. The results showed that the expression levels of the *miR156* were decreased in Col-0 during shoot development and the *g3bp6* mutant had elevated levels of *pri-miR156a* at each of these time points (Figure 5H). *Pri-miR156a* and *pri-miR156c* are the two major sources of mature miR156 for the juvenile-to-adult vegetative phase transition [31,34,35]. We further examined the transcription of miR156a/miR156c and found that only *pri-miR156a* was significantly elevated in *g3bp6*. Additionally, the *G3BP6* itself was also significantly elevated during shoot development (Figure 5H). Collectively, these results suggest that G3BP6 probably represses the level of miR156 by controlling the transcription of *miR156a*, thus regulating downstream targets like SPL proteins during the flowering transition.

### 3.5. Nuclear Localization of G3BP6 Is Essential for Flowering Transition

**Figure 6:**
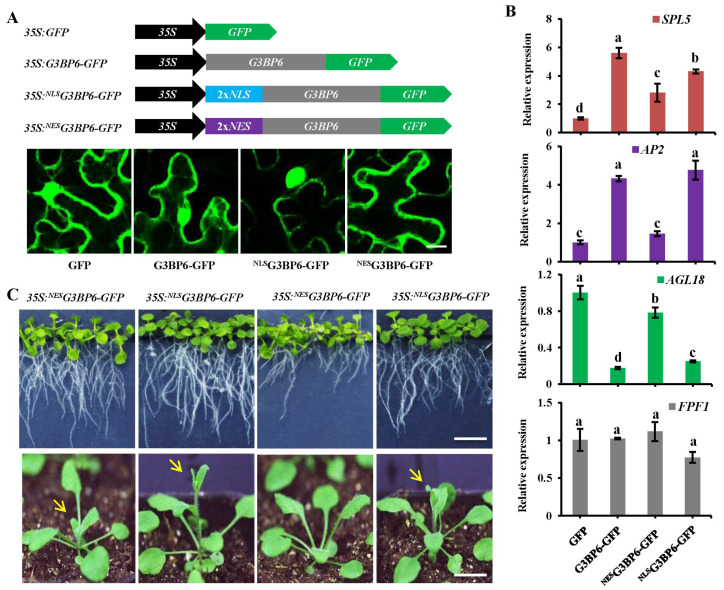
Analysis of the effects of different subcellular pools of G3BP6 for the plant flowering transition. (**A**) Schematics of various forms of G3BP6 and representative images for subcellular localization from confocal microscopy. Scale bar: 50 μm. (**B**) Expression patterns of related genes for flowering via qRT-PCR analysis. Total RNA was extracted from the protoplasts expressed with indicated plasmids and used for reverse transcription. qRT-PCR reactions were run in triplicate and *Actin2* was used as the reference gene. Three biological replications were performed, and similar results were observed. The lowercase letters denote statistically significant differences between the indicated samples, as determined with one-way ANOVA (*p* < 0.05). (**C**) Phenotypic analysis for overexpression of different subcellular pools of G3BP6 in Col-0 plants. The representative two-week- (grown in the solid MS medium; upper panel) and four-week-old (grown in the soil; lower panel) plants are shown. The plant bolting is indicated by arrows. Scale bar: 1 cm.

To testify if the nuclear localization of G3BP6 is important to its function in the flowering transition, we manually changed its subcellular localization by attaching the signal peptides of the NLS and the nuclear export signal (NES) directly to G3BP6 (as ^NLS^G3BP6 and ^NES^G3BP6, respectively) and transiently expressed them in N. benthamiana (Figure 6A). The results revealed that the ^NES^G3BP6-GFP was localized in the cytoplasm, whereas the ^NLS^G3BP6-GFP was mainly localized in the nuclei in leaf epidermal cells. We then compared the effects of the two differently localized G3BP6 variants on the expressions of SPL5, AP2 and AGL18 in Arabidopsis protoplasts. The results showed that the expression of SPL5 was remarkably enhanced when the G3BP-GFP was overexpressed (Figure 6B). Notably, the expression of SPL5 was much higher in cells overexpressing ^NLS^G3BP6-GFP in comparison with that in cells overexpressing ^NES^G3BP6-GFP (Figure 6B). A similar trend was found for the expression of AP2 (Figure 6B). In contrast, the G3BP6-GFP and ^NLS^G3BP6-GFP cells displayed notably repressed AGL18 expression (Figure 6B). To further investigate the role of the nuclear pool of G3BP6 in flowering, we generated transgenic plants overexpressing G3BP6-GFP, ^NES^G3BP6-GFP and ^NLS^G3BP6-GFP on a Col-0 background. In the seedling stage, the plants with G3BP6 and ^NLS^G3BP6 overexpression were similar to that of Col-0 (Figure 6C, upper panel). Unexpectedly, the overexpression of ^NES^G3BP6 led to severe growth defects, such as short root growth. Bolting is an important developmental stage of plants from vegetative growth transit to reproductive growth [36]. We observed that plants with overexpressions of both G3BP6-GFP and ^NLS^G3BP6-GFP bolted much earlier than plants with overexpression of ^NES^G3BP6-GFP (Figure 6C, lower panel). This indicates that the expression of G3BP6 in the nucleus is essential for its role in the flowering transition.

### 3.6. Scaffold Protein RACK1 Contributes to the Nuclear Translocation of G3BP6

**Figure 7:**
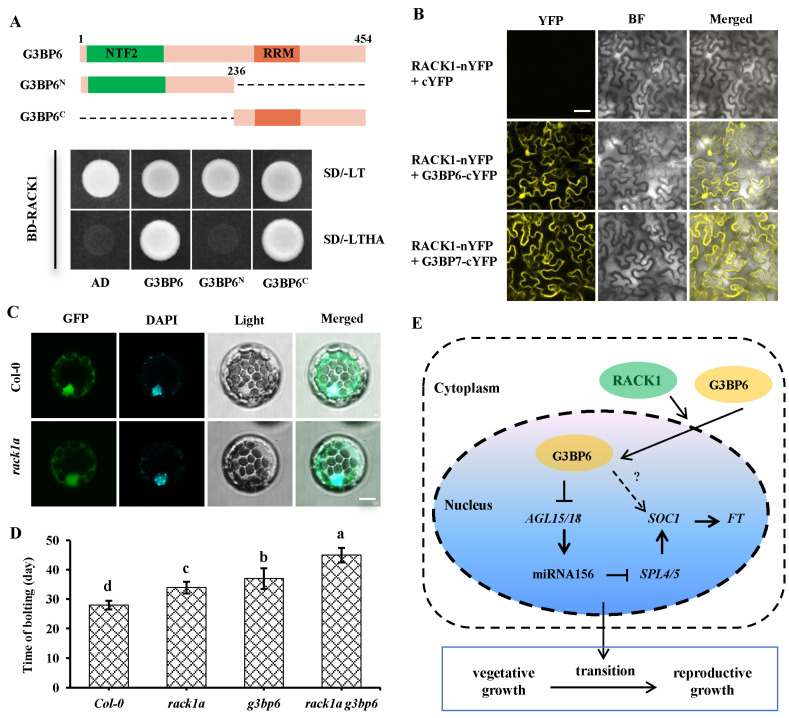
The scaffold protein RACK1 interacts with G3BP6 to regulate the flowering transition. (**A**) Analysis of the interaction between RACK1 and G3BP6 using Y2H. -LT indicates the Leu and Trp drop-out plate, and -LTHA indicates the Leu, Trp, His and Ade drop-out plate. (**B**) In vivo interaction between RACK1 and G3BP6/7, analyzed with BiFC, in N. benthamiana. YFP signals were visualized under confocal microscopy. BF: bright field; scale bar: 50 μm. (**C**) RACK1 increased the nuclear localization of G3BP6. G3BP6-GFP was transformed into the protoplasts from Col-0 or *rack1a* mutant plants and examined under confocal microscopy. Scale bar: 10 μm. (**D**) Analysis of the genetic interaction between RACK1 and G3BP6. The times of blotting from different genetic backgrounds were quantified. The lowercase letters denote statistically significant differences between the indicated samples, as determined with one-way ANOVA (*p* < 0.05). (**E**) A proposed model for RACK1 action on G3BP6 function. RACK1 may contribute to the nuclear translocation of G3BP6 and additively regulate the plant flowering transition.

Since the nuclear localization of G3BP6 is important for its role in plant flowering transition, we asked how the nucleocytoplasmic shutting of G3BP6 is modulated. Screening of the interacting proteins of G3BP6 using the yeast two-hybrid (Y2H) system has identified that the scaffold protein RACK1, which is known to interact with a large number of proteins and modulate the activity and localization of substrates [37], directly interacts with G3BP6 (Figure 7A). To define the domain of G3BP6 that interacts with RACK1, we generated different truncated G3BP6 versions. It was found that the truncated G3BP6 lacking the NTF2-like domain interacts with RACK1, as observed in the Y2H assay. The G3BP6-RACK1 interaction in planta was further verified with a BiFC assay. It was revealed that coexpression with RACK1-nYFP and G3BP6-cYFP in N. benthamiana leaves produces strong YFP fluorescence in both the cytoplasm and the nucleus (Figure 7B). Interestingly, it was found that RACK1 also interacts with G3BP7, which occurred exclusively in the cytoplasm (Figure 7B).

Given that the interaction between RACK1 and G3BP6 occurred both in the cytoplasm and in the nucleus (Figure 7B), we assumed that RACK1 was involved in the nuclear translocation of G3BP6. To test this possibility, we examined the subcellular localization of G3BP6 in Col-0 and the rack1a mutant. When expressed in Col-0, G3BP6 was widely distributed in the cytoplasm and nucleus (Figure 7C). However, the nuclear localization of the G3BP6 was significantly decreased in the protoplasts of the rack1a mutant (Figure 7C). Previous phenotypic analysis has demonstrated that the loss-of-function mutant rack1a has late flowering [38], which is very similar to the delayed-flowering phenotype in g3bp6. We further found additional effects on late flowering in the *rack1a g3bp6* double mutant (Figure 7D). This implies that RACK1 may interact with G3BP6 to regulate the flowering transition.

## 4. Discussion

G3BPs are conserved throughout eukaryotic evolution as members of the heterogeneous nuclear RNA-binding protein family. The roles of G3BPs are well-established in mammals, but their roles in plants are poorly characterized. In this study, we found that loss-of-function mutant *g3bp6* showed a delayed flowering phenotype, whereas the overexpression of G3BP6 significantly promoted bolting, implying that G3BP6 plays a positive role in controlling the plant flowering transition. Moreover, the role of G3BP6 in controlling flowering is highly dependent upon its nuclear localization, which is partially regulated by RACK1, a conserved scaffold protein that has previously been reported to participate in flowering (Figure 7E).

It is well known that G3BPs play important roles in the formation of SGs in mammals. In the present study, we have also shown that most members of the G3BP family from *Arabidopsis* form granule-like structures in the protoplasts, which was much more evident after heat shock treatment, especially for G3BP2, G3BP5 and G3BP6 (Figure 1). These results imply a functional conservancy of G3BPs between mammals and plants. However, it is still largely unclear how the formation of the granule-like structures is mediated by the G3BP family in plant growth and development as well as in responses to environmental stresses. In *Arabidopsis*, there are seven members of the G3BP family, only two members of which have been functionally characterized [15,39]. Loss-of function mutation analysis showed no obvious differences in the growth phenotype when the *G3BPs* were mutated, except for *G3BP6*, which showed a significantly delayed flowering phenotype (Figure 3). At present, we know little about the molecular mechanisms underlying G3BP6 regulation in flowering events.

The flowering process of plants is tightly regulated by a comprehensive molecular network. FLOWERING LOCUS T (FT) and the MADS box gene SOC1 are well-known for their functions in the meristem identity and downstream of the photoperiod, vernalization and autonomous pathways for flowering. Consistently with the delayed flowering in the *g3bp6* mutant, our RNA-sequencing and qRT-PCR assays showed significantly decreased expressions of these two marker genes (Figure 5C,D), whereas the expressions of *AGL15* and *AGL18*, encoding repressors of the floral transition [40,41], were significantly upregulated in the mutant (Figure 5A,E). These results suggest that G3BP6 acts upstream of FT/SOC1 and/or AGL15/18 modules in the signaling pathway that controls flowering. It is well-known that AGL15 and AGL18 positively regulate the expression of *miR156*, thus repressing the downstream SQUAMOSA PROMOTER-BINDING PROTEIN-LIKE (SPL) transcription factors [35,42,43]. Our results showed that the expressions of three members of the SPL family, *SPL4*, *SPL5* and *SPL9*, were significantly downregulated, and the expressions of *SPL13* and *SPL16* were much higher in the *g3bp6* mutant as compared with those in the wild-type Col-0 (Figure 5F,G). Previous studies have shown that SPL3, SPL4 and SPL5 appear to function mostly in the control of flowering time [44]. The overexpressions of SPL3 and SPL9 accelerate flowering, whereas a reduction in SPL activity through miR156 overexpression will delay the onset of flowering [45]. It is reasonable to assume that SPL transcription factor family members are involved in the G3BP6-controlled flowering transition.

G3BPs are generally observed in the formation of SGs under stress, where they coordinate signal transduction with RNA metabolism during the adaptive cellular response. Coincidentally, many miRNAs play key roles in plant flowering by integrating into known age-dependent flowering pathways [46]. Particularly, the miRNA metabolism is required to fine-tune the onset of flowering under fluctuating ambient temperature conditions, implying the association between these miRNAs and G3BPs [47,48]. Among the miRNAs of flowering-time regulation, the highly conserved miRNA156 in land plants is the master regulator of the juvenile-to-adult transition [35,48]. Consistently with the late-flowering phenotype in *g3bp6*, our qRT-PCR analysis revealed significantly higher *miR156* expression, accompanied by significant attenuation of *pri-miR156a* in *g3bp6*, than that in the Col-0 (Figure 5H). Our study has indicated that only the attenuation of *pri-miR156a* is significant in *g3bp6*, implying that G3BP6 specifically regulates the production of *miR156a* to limit the processing of *miR156*. Collectively, these results suggest that G3BP6 may contribute to flowering by affecting the regulatory network of the miR156-SPL module. Human G3BP1 has been shown to regulate the production of *miR-1* from binding to the consensus sequence of the *microRNA (miR)-1-2* precursor and the biogenesis of *microRNA-15b* and *microRNA-23a* [49,50]. It is possible that *Arabidopsis* G3BP6 directly mediates the processing of miR156 or other miRNAs, which is worthwhile for further investigation.

In this study, we found that only G3BP6 was localized both in the cytosol and in the nucleus, whereas the other six G3BPs were mainly localized in the cytosol in *Arabidopsis* (Figure 1D) and the delayed flowering phenotype was exclusively found in the *g3bp6* mutant (Figure 3B). Understandably, the nucleocytoplasmic shuttling of G3BP6 might be a key factor in determining the flowering time of *Arabidopsis*. This is very likely the case, as constitutively expressing the G3BP6 in the cytoplasm led to late flowering in *Arabidopsis*, whereas the nuclear expression of G3BP6 promotes blotting without apparent growth compensation. Importantly, in contrast to human G3BPs, the NTF2-like domain is not necessary for the nuclear localization of G3BP6 in *Arabidopsis* (Figure 2A–C). Instead, the nuclear localization sequence from numbers 237 to 261 of G3BP6 might be responsible for the nuclear localization of G3BP6. Previous studies have shown the roles of RACK1 in the nuclear import of substrates, including the transcription factor Broad Complex (BR-C) from human cells and the brassinosteroid-signaling positive regulator (BZR1) from *Arabidopsis*, as well as in flowering, by regulation of *FT* and *SOC1* [38,51,52]. Our results, showing that the C-terminus of the G3BP6 interacts with RACK1 and with the attenuated nuclear accumulation of G3BP6 in the absence of RACK1, support that RACK1 and G3BP6 may function jointly in regulating the expression of floral genes and ultimately the induction of the flowering process in *Arabidopsis* plants. The detailed mechanisms of G3BP6 and RACK1 in the regulation of plant flowering transitions remain to be further explored.

## Data Availability

Data are contained within the article and Supplementary Materials.

## Supplementary Materials

The following supporting information can be downloaded at: https://www.mdpi.com/article/10.3390/biom13121697/s1, Figure S1: The qRT-PCR analysis for expression patterns of individual *G3BP* genes; Table S1: List of the primers used in this study.
